# Case report: Vitiligo-like toxicity due to ribociclib during first-line treatment of metastatic breast cancer: two cases of premature interruption of therapy and exceptional response

**DOI:** 10.3389/fonc.2023.1067264

**Published:** 2023-03-09

**Authors:** Mariangela Pasqualoni, Armando Orlandi, Antonella Palazzo, Giovanna Garufi, Maria Chiara Cannizzaro, Letizia Pontolillo, Sergio Pannunzio, Claudia Cutigni, Pietro Sollena, Francesco Federico, Emilio Bria, Giampaolo Tortora

**Affiliations:** ^1^ Comprehensive Cancer Center, Medical Oncology Unit, Fondazione Policlinico Universitario ‘A. Gemelli’ – IRCCS, Rome, Italy; ^2^ Catholic University of Sacred Heart, Rome, Italy; ^3^ Department of Dermatology, Fondazione Policlinico Universitario A. Gemelli IRCCS, Rome, Italy; ^4^ Department of Pathology, Fondazione Policlinico Universitario A. Gemelli IRCCS, Rome, Italy

**Keywords:** breast cancer, CDK4/6 inhibitor, skin adverse event, vitiligo, lymphocytic infiltration

## Abstract

Cancer treatment-related adverse events (AEs) are sometimes associated with outcomes for cancer patients, especially with the newest therapies such as target therapy and immunotherapy. A few years ago, the first-line therapy for hormone-receptor-positive metastatic breast cancer (mBC) patients has been deeply changed by the introduction of cyclin-dependent kinase (CDK) 4/6 inhibitors, and now, we are improving our knowledge about their AEs and significance in clinical practice. Here, we report our experience with two cases of vitiligo-like lesions that occur early during treatment with ribociclib. We tried to change the CDK4/6 inhibitor for one patient, but the skin reaction persisted. Both patients retained only the endocrine therapy alone and had an unexpected durable progression-free survival (PFS). Some data on skin toxicities, including vitiligo-like lesions by CDK4/6 inhibitors, have recently been reported in the literature, but for the first time, we highlight a possible correlation with improved survival outcomes of patients. Uncovering the etiology of this toxicity, verifying the involvement of the immune system, and demonstrating a possible positive impact in survival represent an intriguing research objective for the near future.

## Introduction

Breast cancer (BC) is the first leading cause of death for cancer in women. Approximately 20% of BCs spread outside the mammary gland, becoming a metastatic disease, and 70%–80% of these have a Hormone receptor-positive/HER2-negative (HR+/HER2−) immunophenotype (1).

Cyclin-dependent kinases 4 and 6 (CDK4/6) regulate cell-cycle progression, and their overexpression is frequent in luminal BCs; consequently, the inhibition of the pathway consisting of cyclin D, CDK4/6, and retinoblastoma protein is an effective therapeutic strategy for these BC subtypes (2). Therefore, nowadays, the first-line standard of care (SOC) despite age, comorbidities, and the number or sites of metastatic disease is the association of the endocrine therapy with CDK4/6 inhibitors such as ribociclib, palbociclib, and abemaciclib. Thanks to this treatment, the progression-free survival (PFS) of patients with metastatic breast cancer (mBC) is approximately a little more than 2 years with a consistent improvement also for overall survival (OS) (3–5). However, we do not know any predictive biomarkers of response to this treatment yet.

In the literature, there is a known association between treatment toxicity and clinical outcomes for several types of cancer drugs (6). For example, it is well-known how immunologically related adverse events (AEs) predict a good response to immunotherapy (7) or, for BC patients, how post-menopausal symptoms predict improved outcomes among women taking adjuvant endocrine therapy (8).

Pivotal studies of CDK4/6i have shown that the most common cutaneous side effects are mild, with grade 3 rashes only occurring in 0.9% of patients, and at the same time, no evidence of impact on prognosis was shown for skin rash incidence or grade of toxicity (3–5).

Vitiligo is an autoimmune skin disorder that originates from the loss of functional melanocytes of the epidermis, resulting in the appearance of hypopigmented skin areas. In patients affected by malignant melanoma, vitiligo-like lesions occur spontaneously or during anticancer treatments with an incidence that is 10-fold higher than that in the general population (9). Moreover, several studies suggest that the appearance of depigmented patches could be a clinically visible immuno-related event associated with clinical benefit in the context of immunotherapy for melanoma cancer patients (10). In patients with or treated for non-melanoma malignancies, vitiligo-like disorders are absolutely rare.

We discuss two cases of metastatic HR+/HER2− mBC patients treated with the association therapy of CD4/6 inhibitors plus endocrine therapy with a remarkable and durable response after severe skin toxicity and persistent residual diffuse areas of vitiligo-like lesions.

## Case 1

In 2008, a 46-year-old woman was diagnosed with estrogen receptor-positive (80%), progesterone receptor-negative (0%), human epidermal growth factor receptor-2 (HER2)-negative, low-grade (G1) invasive cancer of the right breast (pT2N2, M0; Ki-67 index 2%). Primary treatment was right quadrantectomy and excision of sentinel lymph node, adjuvant chemotherapy (docetaxel and cyclophosphamide for six cycles), irradiation of the affected breast and supra- and infraclavicular nodes, and adjuvant endocrine therapy with tamoxifen for 5 years (until 2013).

Thereafter, the patient remained relapse-free for 13 years, until an MRI was performed due to an accidental trauma and revealed metastases in the whole column. The next contrast-enhanced total-body CT scan confirmed numerous osteolytic lesions in all vertebral metamers and showed metastases also in the sternum and pelvis lymph nodes of 2.7 cm [target lesion according to the Response Evaluation Criteria in Solid Tumors (RECIST) v.1.1 criteria]. The patient’s Eastern Cooperative Oncology Group Performance Status (ECOG PS) was 0, and she did not have any comorbidities.

**Figure 1:**
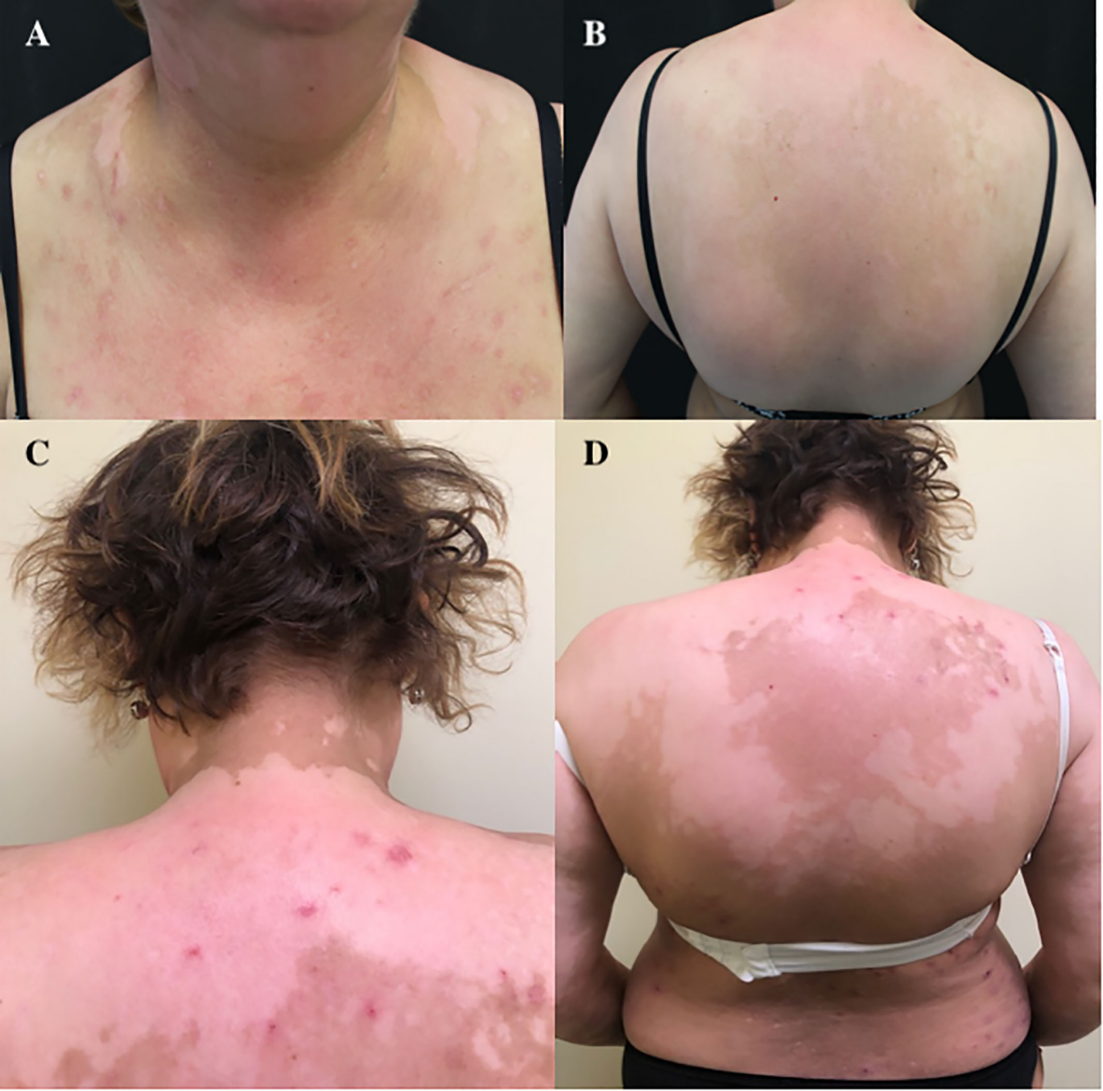
**(A)** Erythematous-vesicular cutaneous rash after 3 months of therapy with ribociclib. **(B–D)** Residual depigmented areas of the back.

In consideration of the recurrence sites, it was decided not to perform a biopsy, and according to the history of the patient, in July 2017, she started a systemic therapy with a CDK4/6 inhibitor, ribociclib 600 mg/day (three 200-mg tablets daily for 3 weeks every 4 weeks), plus endocrine therapy with letrozole (2.5 mg/day) as the SOC for HR+/HER2− metastatic BC patients (3–5). After 3 months of therapy, the patient developed itchiness followed by a diffuse erythematous-vesicular cutaneous rash in certain areas that evolved into leukodermic lesions (on the trunk, legs, and both arms) (**Figure 1A**). Thus, we primarily performed a dose reduction of ribociclib (400 mg/day, two 200-mg tablets daily for 3 weeks every 4 weeks) in association with oral (dexamethasone) and topical (clobetasol) steroids. Despite our change in therapy, the cutaneous toxicity did not resolve; thus, after another cycle of therapy with dose-reduced ribociclib (400 mg/day), the use of systemic antineoplastic drugs was stopped, retaining only the endocrine therapy. After 3 months without a cyclin-CD4/6 inhibitor, the cutaneous rash and itchiness disappeared, but depigmentation areas remained (**Figure 1B**).

**Figure 2:**
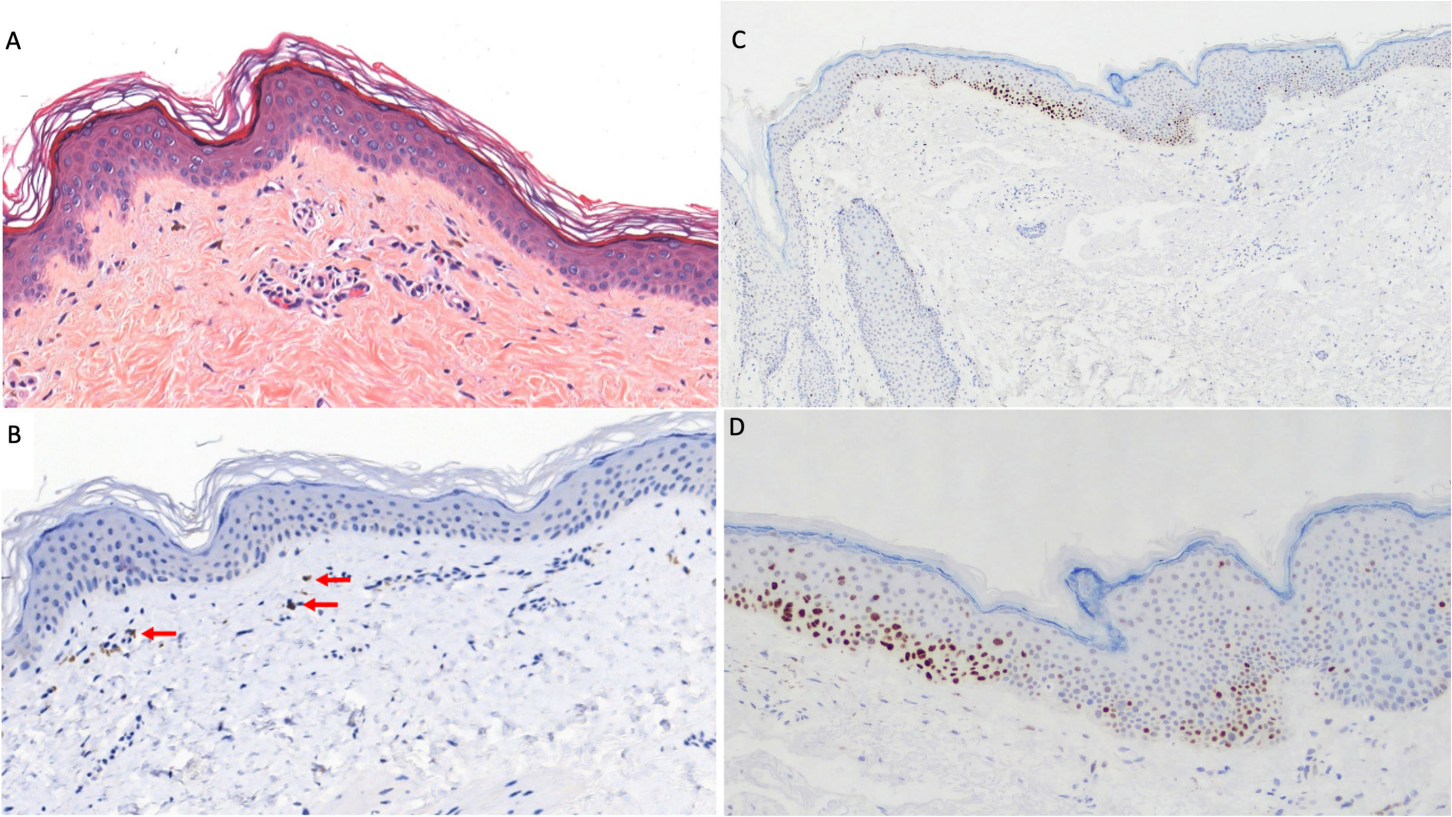
Skin histopathologic and immunohistochemical examination. **(A)** Mild perivascular lymphocytic infiltrate in the superficial dermis extended along the basal layer (hematoxylin–eosin, original magnification ×100). **(B)** Focally detected melanin (red arrows) in few dermal melanophages (melan-A monoclonal antibody, A103, Invitrogen; original magnification ×100). **(C, D)** Strong P53 expression (mouse monoclonal anti-P53 antibody MS–738-R7-LabVision/Neomarkers, USA) in basal and supra-basal layers in depigmented skin at original magnification ×50 **(C)** and ×100 **(D)**.

A histopathologic examination of a skin biopsy specimen was performed and showed a mild lymphocytic infiltrate (CD8+) along the basement membrane layer (**Figure 2A**). The Fontana Masson staining test was negative with no single melanocytes and few melanophages in the dermis (**Figure 2B**). Also, P53 expression was determined using immunohistochemical analysis with evidence of strong immunostaining in basal and supra-basal layers in depigmented skin (**Figures 2C, D**).

During the treatment, the patient underwent contrast-enhanced total-body CT scans every 3–4 months, and the best response documented was a stable disease per RECIST v1.1. Furthermore, all osteolytic bone metastases became blastic bone metastases.

Accordingly, owing to the good response of cancer to the association therapy despite the toxicity, we tried to give the patient another CDK4/6 inhibitor, palbociclib (125 mg/day, one 125-mg tablet daily for 3 weeks every 4 weeks), with letrozole. After a few days of the first cycle, the patient again developed an erythematosus cutaneous rash; thus, we immediately stopped the therapy. The patient underwent treatment with antihistamine (ebastine) and topical steroid (clobetasol) therapy, thus controlling the cutaneous rash.

Currently, the patient is still taking endocrine therapy alone with letrozole (ongoing at the time of writing), and she has persistent and extensive vitiligo-like lesions (**Figures 1C, D**), but she remains in good clinical condition. She will be followed up with a regular CT scan every 3–4 months.

The last radiological evaluation was performed in November 2021, and it confirmed a persistent response to our treatment, with a PFS of more than 4 years (50 months) despite taking the CDK4/6 inhibitor for a few months, which compares favorably with the median PFS of 25.3 months with the combination therapy in the Phase 3 MONALEESA-2 study (5).

## Case 2

An 80-year-old woman has a history of early BC ER/PR-positive, HER2-negative diagnosed in 1995 (pT2, pN0, M0) for which she underwent a right quadrantectomy and homolateral axillary lymphadenectomy followed by adjuvant radiotherapy and then endocrine therapy with tamoxifen for 5 years according to her premenopausal state. Ten years after surgery, the patient has had a local recurrence of the disease in the upper external right quadrant for which a right upper quadrantectomy was performed in 2005 (ductal invasive BC ER/PR-positive, HER2-negative, grade 1; pT1c). After that, hormonal therapy with anastrozole (1 mg/day) was started according to her post-menopausal state for 5 years, until 2010.

In 2017, because of the high values of tumor markers CEA and CA15.3, the patient underwent a CT scan that documented multiple lung and liver metastases and mediastinal lymph nodes confirmed by the next PET/CT with 18-FDG. The ECOG PS was 0 and she did not have any comorbidities. Therefore, according to her oncological history, in October 2017, she started a first-line endocrine therapy with letrozole (2.5 mg/day) plus CDK4/6i ribociclib (400 mg/day, two 200-mg tablets daily for 3 weeks every 4 weeks).

**Figure 3:**
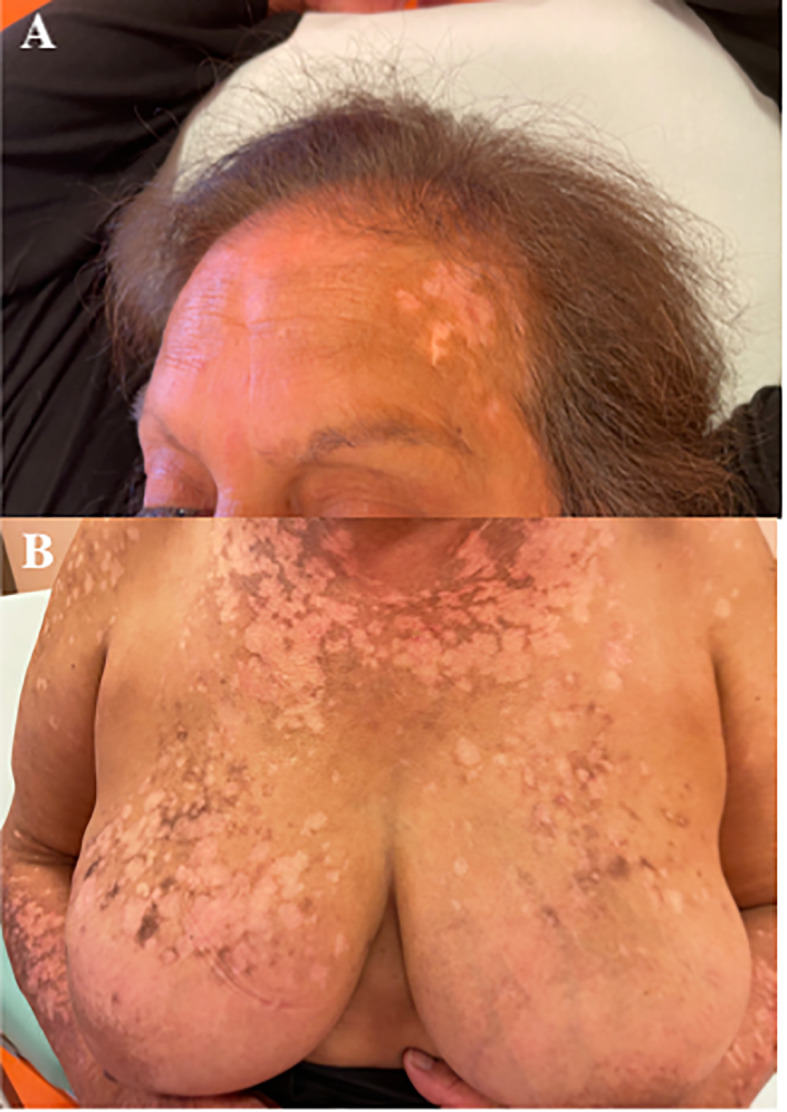
**(A, B)** Extensive depigmentation cutaneous areas after 1 month of stopping the therapy with ribociclib.

After 12 months of treatment, the patient developed itchiness and then a diffuse erythematous-vesicular cutaneous rash similar to our first case; this was followed by some areas evolving into permanent depigmented lesions. Thus, the use of systemic antineoplastic drugs was stopped, retaining only letrozole. After 1 month without ribociclib, the cutaneous rash and itchiness disappeared, but several extensive depigmentation areas remained (**Figures 3A, B**). Despite the attempt to resume treatment with a reduced dose of ribociclib (at both 400 and 200 mg/day), the rash with widespread itching recurred, and therefore, it was decided to permanently discontinue ribociclib and to continue letrozole alone.

During the treatment, the patient underwent CT scans every 3–4 months, and the best response documented was stable disease. In October 2021, the patient underwent a PET/CT-18FDG that documented a persistent complete metabolic response to the treatment with a median PFS of 39 months despite the discontinuation of combination therapy.

## Discussion

HR+/HER2− is the subtype of mBC with a better survival outcome thanks to the first-line use of CDK4/6 inhibitors with endocrine therapy (3–5). We reported two cases of HR+/HER2− mBC patients who achieved long-term stabilization of the disease with endocrine therapy alone after approximately only a few months with the SOC in the first-line setting, which is endocrine therapy with ribociclib.

Usually, CDK4/6 inhibitors are well tolerated and AEs are typically easily managed with dose modification and supportive care measures. Without a comparative study between the different possible combinations of palbociclib, ribociclib, and abemaciclib in first- and second-line therapy, the choice of physicians is currently mainly driven by the different toxicity profiles of these drugs. The most common AE reported with palbociclib and ribociclib is neutropenia, while the most common AE reported with abemaciclib is diarrhea. Treatment discontinuation was significantly higher with abemaciclib than with palbociclib, but similar between ribociclib and palbociclib. Nowadays, cutaneous AEs are not taken into consideration when choosing what CD4/6 inhibitor to give to the patient. Nevertheless, real-world data have shown that skin toxicity could reduce the tolerability of the therapy for our BC patients, leading to a 25% discontinuation rate (11). Alopecia is the most frequent dermatological AE (7%–33%) of all CD4/6 inhibitors, and it has occurred after approximately 3–4 months of treatment. Other frequent dermatological AEs are maculopapular rash and pruritus, which are commonly mild in severity and more frequent with ribociclib than with palbociclib and abemaciclib. Palbociclib was more associated with dry skin and onychoclasis (11).

We know that cutaneous toxicities diminish the quality of life (QoL) of patients, which impacts their treatment adherence and can affect their personal, social, and workplace relationships, jeopardizing the treatment's success and patient survival (12). Notably, their frequency and severity may be associated with clinical benefit from anticancer therapies; thus, mitigating these events is of importance to maintaining dose intensity and QoL.

The patients in our case reports have had pruritus and an extensive cutaneous rash, the first one after 2 months of therapy and the second one after 1 year. Due to this event, both patients stopped the treatment with CDK4/6 inhibitors, but in both cases, they developed persistent skin-depigmented vitiligo-like areas.

Cancer patients treated with anti‐PD1/PD-L1 therapies as well as with tyrosine kinase inhibitors (imatinib, cabozantinib, and pazopanib) experience common skin immune-related AEs such as vitiligo-like lesions, rash, and pruritus (13–15). Moreover, vitiligo occurs in approximately 20% of melanoma patients, which is higher than that of the general population (0.5%–1%), which could be associated with better clinical response and survival (15). It probably occurs due to the immune activation against melanoma-associated antigens expressed by normal melanocytes (MART-1 and gp-100) as a result of cross-reaction from melanoma cells that share the same antigens (16).

Our patients have had very good outcomes despite their skin AEs, with a median PFS that is more similar to the typical value of the combination therapy than that of the endocrine therapy alone. We can speculate about the early onset and/or the severity of the skin AEs in our patients and their eventual prognostic role as well as the correlation between our patients’ persistent response to the therapy.

In 2020, Sollena et al. reported an international retrospective study including patients with advanced BC who developed vitiligo-like lesions during treatment with CDK4/6i. Of the 16 patients included in this study, 3 have undergone a cutaneous biopsy of the hypopigmented areas, resulting in the detection of a mild lymphocytic infiltrate along the basement membrane layer with few melanophages in the dermis and a negative Fontana staining (17). These results are similar to what we have found in our patient. In one case, we tried to change the treatment by switching from ribociclib to palbociclib without success. It is possible that the mechanism behind this skin AE is class related.

One possible mechanism to explain this rare skin reaction to CDK4/6 inhibitors is hypothesized by Sollena et al.: that alterations in keratinocyte precursor proliferation and the apoptosis induced by CDK4/6i may lead to a loss of survival stimuli and passive melanocyte premature death, with the consequent onset of hypopigmented lesions (17). This hypothesis is consistent with the results of the histological examination of the skin in the first patient reported in this case series. In fact, we observed evidence of signs of apoptosis in basal and supra-basal layers (P53+), a lymphocytic infiltrate CD8+, and hypopigmentation with few melanophages in the dermis (Fontana staining negative).

At the same time, an intriguing immune-mediated mechanism may justify a correlation between the irreversible vitiligo-like skin toxicity and the exceptional response with limited exposure to CDK4/6 inhibitors. Skin toxicity of CDK4/6 inhibitors, in addition to inducing a passive death of melanocytes, could determine an activation against BC-associated antigens expressed by normal melanocytes. As a result of cross-reaction from melanocytes that could share some antigens also expressed by BC (18), an immune reaction could occur that feeds and makes vitiligo-like toxicity irreversible and, at the same time, helps control the metastatic disease. This hypothesis is also supported by the presence of lymphocyte infiltrates in the skin biopsy performed in the first case report, but other *in vitro* and *in vivo* studies are needed to verify this theory and better understand this peculiar toxicity of these new drugs.

In conclusion, these two case reports documented response to endocrine therapy alone after a premature interruption of CDK4/6i due to a severe cutaneous AE with a vitiligo-like irreversible outcome in two patients with metastatic HR+/HER2− BC that, at the time of last follow-up, both have a PFS of approximately 40 months. Nowadays, the prognostic value of AEs and especially vitiligo-like toxicity in patients treated with CDK4/6i remains unknown and warrants further investigation.

## Data availability statement

The original contributions presented in the study are included in the article/supplementary material. Further inquiries can be directed to the corresponding author.

## Ethics statement

Ethical approval was not provided for this study on human participants because the approval of the ethics committee is not required for the collection of clinical cases in Italy. The patients/participants provided their written informed consent to participate in this study. Written informed consent was obtained from the individual(s) for the publication of any potentially identifiable images or data included in this article.

## Author contributions

AP, GG, LP, MC, SP and CC were involved in the care of the patients. MP and AO wrote the final version of the manuscript. PS and FF made the histological images and their descriptions. All authors contributed to the article and approved the submitted version.
